# Evaluation of the inhibitory effect of the cell-free fermentation filtrate of *Bacillus atrophaeus* YL84 on *Fusarium oxysporum* f. sp. *vasinfectum* and analysis of its metabolic products

**DOI:** 10.3389/fmicb.2026.1761389

**Published:** 2026-01-22

**Authors:** Yuxin Tang, Zhe Wang, Hongzu Feng, Lan Wang

**Affiliations:** 1 Laboratory of Green Pest Management, College of Agriculture, Tarim University, Aral, China; 2 Key Laboratory of Integrated Pest Management in Southern Xinjiang, Aral, China

**Keywords:** *Bacillus atrophaeus*, biological control, cell-free fermentation filtrate, cotton *Fusarium* wilt, UHPLC-IM-QTOF-MS

## Abstract

To evaluate the biocontrol potential of the cell-free fermentation filtrate (CFFF) of *Bacillus atrophaeus* strain YL84 against *Fusarium oxysporum* f. sp. *vasinfectum* (FOV), this study systematically investigated the effects of the CFFF at various dilution ratios on FOV mycelial growth, conidial germination, cellular nucleic acid leakage, and malondialdehyde (MDA) content. Furthermore, the environmental stability of its antifungal activity was assessed. In addition, a dual-culture assay was conducted to evaluate the antagonistic activity of strain YL84 against FOV. The results of the dual-culture assay showed that strain YL84 significantly inhibited the growth of FOV, with an inhibition rate of 81.06%. Subsequently, the YL84 CFFF exerted significant inhibitory effects on FOV mycelial growth and conidial germination across different concentrations, achieving maximum inhibition rates of 75.68% and 77.56%, respectively. Notably, the treated mycelia exhibited a significant increase in cellular nucleic acid leakage and elevated levels of MDA, a product of lipid peroxidation, suggesting that the CFFF may disrupt the integrity of the pathogen’s cell membrane. Stability assays revealed that the CFFF possessed substantial tolerance to high temperatures, ultraviolet irradiation, and hypersaline environments, although it remained sensitive to strongly alkaline conditions. Greenhouse pot experiments further confirmed the efficacy of YL84 CFFF in controlling cotton *Fusarium* wilt, with a maximum control efficacy of 69.21%. Moreover, the treatment induced the upregulation of defense-related enzyme activities in the plants, suggesting that the CFFF may function through both direct antifungal action and the elicitation of host-induced resistance. Component identification *via* Ultra-Performance Liquid Chromatography–Ion Mobility–Quadrupole Time-of-Flight Mass Spectrometry (UPLC-IMS-Q-TOF-MS) suggested that the filtrate is rich in structurally diverse compounds that were putatively identified as potential antimicrobial substances, predominantly classified as terpenoids and their derivatives. In conclusion, this study provides a systematic evaluation and supporting evidence for the further development of *B. atrophaeus* YL84 as a biocontrol agent.

## 1 Introduction

Cotton (*Gossypium hirsutum* L.) is one of the world’s most important economic crops, and China ranks among the leading producers globally (Ma et al., 2024; Zhang C. W. et al., 2024; Zhang T. et al., 2024). According to the United States Department of Agriculture (USDA), global cotton production reached approximately 25.35 million tons in 2024, with China contributing 6.05 million tons, highlighting its critical role in the global textile industry. The Xinjiang Autonomous Region is the largest cotton-producing area in China, accounting for 84.93% of the national planting area and 90.99% of the total production as of 2024 (Wang C. et al., 2025; Wang T. et al., 2025). As a principal economic crop, cotton constitutes a vital income source for farmers and local governments, providing strong support for regional development (Wang J. et al., 2022; Wang X. et al., 2022). Cotton *Fusarium* wilt, a devastating soil-borne disease primarily affecting seedlings, is caused by *Fusarium oxysporum* f. sp. *vasinfectum* (FOV) (Wagner et al., 2021). Seedlings are highly susceptible to adverse conditions and pathogens; infection at this stage severely impairs growth and can lead to extensive mortality. If the disease progresses into middle or late growth stages, it triggers large-scale square and boll abscission, reduces fiber length and strength, and ultimately compromises yield and quality (Abdelraheem et al., 2024; Asif et al., 2023; Davis et al., 2023; Dai, 2024). Current management strategies rely mainly on crop rotation, resistant cultivars, and biological control. While rotation and resistant varieties can be effective, breeding programs are time-consuming, and rotation is often impractical due to cropping system constraints (Munir et al., 2016; Shaheen et al., 2021). Chemical fungicides offer partial control, but their prolonged use risks selecting for resistant pathogen populations and can adversely affect soil ecosystems (Jin et al., 2021; Zhang et al., 2017). Therefore, biological control represents a promising, environmentally friendly, and sustainable alternative for plant disease management (Miljaković et al., 2020; Kong et al., 2022).

Studies worldwide have demonstrated that various microorganisms can antagonize FOV effectively, including *Bacillus* spp. (Yang et al., 2024), *Streptomyces* spp. (Abdelghany et al., 2024), *Trichoderma* spp. (Kumari et al., 2024), and *Pseudomonas* spp. (Abo-Zaid et al., 2023). *Bacillus* species have been extensively investigated owing to their strong adaptability, diverse modes of action, and low tendency to induce pathogen resistance (Madriz-Ordeñana et al., 2022). For instance, Sun et al. (2023) reported that *Bacillus velezensis* BVE7, isolated from the soybean rhizosphere, significantly inhibited *F. oxysporum* mycelial growth and spore germination. By enhancing host defense enzymes, it achieved 75.13% control of soybean root rot in pot trials, demonstrating high biocontrol potential. Wang J. et al. (2022) and Wang X. et al. (2022) found that *Bacillus amyloliquefaciens* NCPSJ7 produced the lipopeptide C14-iturin A, which disrupted fungal cell walls and membranes, increased permeability, impaired organelle function, and strongly suppressed mycelial growth and spore germination. Jia et al. (2021) isolated *Bacillus subtilis* BS06 *via* plate confrontation from the soybean rhizosphere; greenhouse trials showed that BS06 not only suppressed *F. oxysporum* infection but also promoted soybean growth while controlling root rot. Although antagonistic bacteria secrete antimicrobial peptides, lipopeptides, enzymes, and antibiotics that effectively inhibit pathogen growth (Ajuna et al., 2024), the stability of these metabolites under field conditions is often compromised by environmental factors such as temperature, pH, and enzymatic degradation, limiting their practical efficacy (Wu et al., 2018).

Despite reports of multiple antagonistic strains against FOV, the available diversity remains insufficient to meet practical needs. Moreover, comprehensive studies evaluating the stability and composition of inhibitory metabolites are still scarce, leaving a significant knowledge gap in translating laboratory findings into robust field applications consequently, there is a pressing need to discover new, effective *Bacillus* strains and to comprehensively evaluate their inhibitory metabolites for the sustainable management of cotton wilt. *Bacillus atrophaeus* YL84, previously isolated in our laboratory from healthy pear leaves, exhibits strong antagonistic activity against several phytopathogens, including *Verticillium dahliae* and *Cytospora pyri*. To address the aforementioned gaps, this study aimed to: (1) assess the antagonistic activity of YL84 against FOV and the inhibitory effect of its CFFF, (2) evaluate the stability of the inhibitory substances under various environmental stresses, (3) conduct a preliminary, putative identification of the antimicrobial constituents in the YL84 CFFF using UPLC-IMS-Q-TOF-MS. This work aims to provide a high-quality microbial resource for the green control of FOV and to lay a theoretical foundation for developing efficient and durable biocontrol formulations.

## 2 Materials and methods

### 2.1 Media and strains

The test pathogen, *Fusarium oxysporum* f. sp. *vasinfectum* (FOV), and the antagonistic strain, *Bacillus atrophaeus* YL84, were provided by the Laboratory of Green Control, Tarim University. The FOV strain was originally isolated from the roots of diseased cotton plants collected from infected cotton fields in Xinjiang, identified based on morphological characteristics and ITS sequence analysis, and subsequently preserved at the Laboratory of Green Control, Tarim University, and its pathogenicity was confirmed *via* Koch’s postulates (Dai, 2024). Strain YL84 was originally isolated from the leaves of Korla fragrant pear using the serial dilution method and identified based on morphological observation, physiological and biochemical characteristics, and 16S rDNA phylogenetic analysis (Tang Y. X. et al., 2025; Tang Y. et al., 2025).

The culture media used in this study are listed in Table 1.

**Table 1:** Culture media used in the experiment.

Medium	Composition
Potato dextrose agar medium (PDA)	Potato 200.0 g, glucose 20.0 g, agar 18.0 distilled water1,000 mL
Potato dextrose broth (PDB)	Potato 200.0 g, glucose 20.0 g, distilled water 1,000 mL
Luria-Bertani (LB)	Tryptone10.0 g, yeast extract5.0 g, NaCl 5.0 g, agar 18 g, distilled water 1,000 mL
LB broth	Tryptone 10.0 g, yeast extract 5.0 g, NaCl 5.0 g, distilled water 1,000 mL

### 2.2 Antagonistic activity of strain YL84 against FOV

Strain YL84 was activated by streaking onto LB agar plates and incubating at 28 °C in the dark for 48 h. FOV was activated on PDA plates at 27 °C in the dark for 5 d (This incubation period was selected because FOV typically exhibits vigorous mycelial growth and fully colonizes the PDA plate within 5 days, which is suitable for evaluating mycelial growth inhibition in dual-culture assays.). The antagonistic activity was assayed using the dual-culture plate confrontation method. A 5-mm diameter mycelial plug of FOV was inoculated at the center of a fresh PDA plate. Activated YL84 colonies were spot-inoculated at four symmetrical points, each 30 mm from the plate center. Plates inoculated with FOV plugs alone served as controls. Each treatment was performed in triplicate. The plates were incubated at 27 °C in the dark until the mycelia in the control group covered the entire plate. Colony diameters were measured using the cross method, and the inhibition rate was calculated using the following formula:


$$\begin{array}{l} \text{Inhibition rate}\;(\%)=(\begin{array}{l} \text{diameter of control colony}\\ -\text{diameter of treated colony} \end{array})\\ /(\begin{array}{l} \text{diameter of}\;\\ \text{control colony} \end{array})\times 100. \end{array}$$

### 2.3 Inhibitory effect of YL84 CFFF on FOV spore germination

After culturing FOV on PDA plates at 27 °C in the dark for 10 d (The longer incubation period was used to allow sufficient conidial production, as FOV produces abundant and mature conidia after prolonged cultivation.), the plates were rinsed with 10 mL of sterile water. The resulting suspension was filtered through double-layered sterile gauze to obtain a conidial suspension, which was quantified using a hemocytometer and diluted with sterile water to 1 × 10^7^ spores mL^−1^. Strain YL84 was inoculated into 100 mL of LB liquid medium and incubated at 28 °C with shaking at 180 r/min for 60 h. The fermentation broth was centrifuged at 12,000r/rmin for 10 min at 4 °C, and the supernatant was filtered through a 0.22 μm membrane to obtain the CFFF.

Following the method of Tang Y. X. et al. (2025) and Tang Y. et al. (2025), the CFFF was used undiluted (stock) or diluted 10-, 100-, and 1,000-fold. An equal volume of each CFFF dilution was mixed thoroughly with the spore suspension (1 × 10^7^ spores mL^−1^). A 100 μL aliquot of the mixture was placed onto a concave microscope slide. A mixture of equal volumes of sterile water and spore suspension served as the control. Each treatment was replicated three times. After incubation at 27 °C in a moist chamber in the dark for 36 h, a time point chosen to allow conidial germination while minimizing extensive hyphal elongation, spore germination was observed under an optical microscope (germination was defined as a germ tube length exceeding half the spore diameter). To ensure that the results reflected germination inhibition rather than early hyphal growth, only the presence/absence of germ tube emergence was recorded, whereas subsequent hyphal elongation was not included in the assessment. The spore germination inhibition rate was calculated as follows:


$$\begin{array}{l} \text{Spore germination inhibition rate}\;(\%)=(\begin{array}{l} \text{germinated spores}\\ \text{in control}-\text{germinated}\;\\ \text{spores in treated} \end{array})\\ /\text{germinated spores}\;\\ \text{in control}\times 100. \end{array}$$

### 2.4 Inhibitory effect of YL84 CFFF on FOV mycelial growth

The mycelial growth rate method was employed as described by Tang Y. X. et al. (2025) and Tang Y. et al. (2025). YL84 CFFF was added to 100 mL of molten PDA medium (cooled to approximately 60 °C) to achieve final volume fractions of 2, 4, 6, 8, and 10%. The mixtures were homogenized and poured into Petri dishes. PDA plates without CFFF served as controls. A 5-mm diameter FOV mycelial plug was inoculated at the center of each plate. Treatments were performed in triplicate and incubated at 27 °C in the dark until the control colonies covered the plate. Colony diameters were measured using the cross method, and the inhibition rate was calculated using the formula described in Section 2.2.

### 2.5 Effect of YL84 CFFF on nucleic acid leakage and malondialdehyde (MDA) content in FOV

One milliliter of FOV spore suspension (1 × 10^7^ spores mL^−1^) was inoculated into 100 mL of PDB and incubated at 27 °C with shaking at 120 r/min for 5 d. Mycelia were collected by vacuum filtration, rinsed three times with 0.2 mol/L phosphate-buffered saline (PBS, pH 7.2), and resuspended in sterile water. Equal amounts of wet mycelia were aliquoted into 1.5 mL centrifuge tubes and treated with 200 μL of undiluted, 10-fold, 100-fold, or 1,000-fold diluted CFFF. An equal volume of sterile water was added for the control. Each treatment was replicated three times.

*Nucleic acid leakage*: Following the method of Pan et al. (2022) mycelia were removed by filtration at 2, 4, 6, and 8 h post-treatment. The absorbance of the filtrate was measured at 260 nm, serving as an index of relative nucleic acid leakage.

*MDA content*: Following the method of Zhang et al. (2020), mycelia were collected by filtration 24 h post-treatment. A 0.5 g sample of wet mycelia was homogenized in an appropriate volume of PBS (0.2 mol/L, pH 7.2) in an ice bath. The MDA content was determined using an MDA assay kit (Thiobarbituric acid method).

### 2.6 Stability assessment of antimicrobial activity of YL84 CFFF

*pH Stability*: Ten milliliters of CFFF were adjusted to pH 3.0, 5.0, 7.0, 9.0, and 11.0 using 1 mol L^−1^ HCl or NaOH, incubated at room temperature for 1 h, then readjusted to pH 7.0.

*Temperature stability*: Ten milliliters of CFFF were treated in water baths at 20, 40, 60, 80, and 100 °C for 30 min and immediately cooled in an ice bath for 5 min.

*UV stability*: Ten milliliters of CFFF in sterile tubes were irradiated with a 30 W UV lamp positioned 30 cm above for 10, 20, 30, 40, or 50 min.

*NACL tolerance*: Five milliliters of CFFF were mixed with equal volumes of 2, 4, 6, 8, or 10% (w/v) NaCl solutions to achieve final concentrations of 1%–5% and incubated for 3.5 h.

### 2.7 Pot experiment for control efficacy of YL84 CFFF against cotton *Fusarium* wilt

Plump seeds of cotton cultivar “Tahe 2” were surface-sterilized with 75% ethanol for 30 s, air-dried, and sown in potting soil. The pots were filled with sterilized nutrient soil mixed with vermiculite at a volume ratio of 2:1. When seedlings reached the two-true-leaf stage, they were inoculated using the root-wounding method: roots were lightly scratched with a sterile scalpel and irrigated with 10 mL of FOV spore suspension (1 × 10^7^ spores mL^−1^). Three days after pathogen inoculation, CFFF treatments were applied by root irrigation. Specifically, each seedling was irrigated with 10 mL per plant of undiluted, 10-fold, 100-fold, or 1,000-fold diluted CFFF.

Irrigation with an equal volume of sterile water served as the control. Each treatment was replicated three times with five seedlings per replicate. All pots were maintained in a greenhouse under controlled conditions at 24 °C, with approximately 80% relative humidity and a photoperiod of 16 L/8 D. Disease severity was assessed 21 d after CFFF treatment to calculate control efficacy.

Cotton *Fusarium* wilt grading standard (Grade 0–IV):

Grade 0: *Healthy* plant, no symptoms.
Grade I: < 25% of *leaves* show yellow vein netting or yellowing, reddening, or purpling.
Grade II: 25%–50% of leaves show symptoms; slight plant stunting.
Grade III: > 50% of leaves show symptoms; obvious plant stunting.
Grade IV: Severe leaf *withering* and abscission; stem death or acute whole-plant wilt and death.

$$\text{Disease index}=[\sum (\begin{array}{l} \text{Number}\;\text{of diseased}\;\\ \text{plants}\;\mathrm{at}\;\text{each level}\\ \times \text{Representative value} \end{array})]/(\begin{array}{l} \text{Total number of}\\ \;\text{plants}\times \text{Highest}\;\\ \text{representative value} \end{array})\times 100\%.$$

$$\begin{array}{l} \text{Control efficacy}\;(\%)=(\begin{array}{l} \text{disease}\;\text{index}\;\\ \text{of control}-\text{disease}\;\\ \text{index of treatment} \end{array})\\ /\text{disease index}\;\\ \text{of control}\times 100. \end{array}$$

### 2.8 Induction of resistance in cotton by YL84 CFFF

In the pot experiment, 2–5 g of cotton leaves were collected from each treatment 21 d after FOV inoculation, flash-frozen in liquid nitrogen, and stored at −80 °C for the determination of defense-related enzymes. Leaf tips and veins were removed, and 2.0 g of the middle-upper leaf tissue was homogenized in an ice bath with 5.0 mL of 0.1 mol/L phosphate buffer (pH 7.2) and a small amount of quartz sand. The homogenate was transferred to a centrifuge tube, mixed thoroughly for 5 min, and centrifuged at 3500 r/min for 10 min at 4 °C. The supernatant served as the crude enzyme extract. Enzyme activities were measured using plant enzyme assay kits obtained from Solarbio Science and Technology Co., Ltd. (Beijing, China).

### 2.9 Composition analysis of YL84 CFFF

Non-targeted metabolomic profiling was performed using Ultra-Performance Liquid Chromatography–Ion Mobility–Quadrupole Time-of-Flight Mass Spectrometry (UPLC-IMS-Q-TOF-MS). A 200 μL aliquot of YL84 CFFF (pre-filtered through a 0.22 μm membrane) was placed on ice. Three volumes (600 μL) of pre-cooled chromatographic-grade methanol were added, vortexed, and incubated on ice for 10 min to precipitate proteins. The mixture was centrifuged at 14,000 r/min for 10 min at 4 °C. The supernatant was collected, filtered through a 0.22 μm filter, and concentrated to near dryness under a gentle stream of nitrogen in a vacuum concentrator. The residue was reconstituted in 1000 μL of the initial mobile phase [positive ion mode: water–acetonitrile (95:5, v/v) containing 0.1% formic acid; negative ion mode: water–acetonitrile (95:5, v/v)], vortexed until homogeneous, and transferred to a vial for UPLC-IMS-Q-TOF-MS analysis.

Chromatographic separation was carried out on a Waters ACQUITY UPLC BEH C18 column (50 mm × 2.1 mm, 1.7 μm). The column temperature was maintained at 35 °C, and the autosampler was set to 10 °*C. mobile* phase A consisted of 0.1% formic acid in water (positive mode) and mobile phase B was acetonitrile containing 0.1% formic acid. The elution gradient was as follows: initial 5% B; 0–27 min, linear increase from 5% to 100% B; 27–28 min, hold at 100% B; 28–28.1 min, rapid return to 5% B; 28.1–30 min, re-equilibration at 5% B. The flow rate was constant at 0.3 mL/min, and the injection volume was 2 μL.

Mass spectrometric data were acquired using a Waters VION IMS Q-TOF mass spectrometer equipped with an electrospray ionization (ESI) source and TWAVE ion mobility technology. Full scans were performed independently in both positive and negative ion modes. Key parameters were: capillary voltage, 2.5 kV (positive) and 2.5 kV (negative); source temperature, 120 °C; desolvation gas temperature, 400 °C; desolvation gas flow, 600 L/h; and cone gas flow, 50 L/h. The full scan mass range was set to *m/z* 50–2000, and the acquisition mode was data-independent MSE. Leucine enkephalin solution was used as the lock mass for real-time correction to ensure mass accuracy.

Raw data were processed using UNIFI software (Waters Corporation) to extract total ion chromatograms (TIC). Compound identification involved the following steps: First, accurate molecular masses from MS1 spectra were matched against public databases (e.g., natural product databases, HMDB) and an in-house microbial secondary metabolite library (mass error tolerance < 5 ppm). MS/MS spectra were compared with literature reports to assist structural confirmation. Finally, the top ten representative active components with high content and distinct antifungal potential were screened based on three core principles: mass spectral response intensity (relative abundance), known antifungal bioactivity, and chemical structural diversity, integrating data from both positive and negative ion modes.

### 2.10 Statistical analysis

Each experiment was performed with at least three repetitions. Visual representations were produced utilizing GraphPad Prism 8. Data processing was carried out *via* SPSS 27.0. Before implementing Duncan’s multiple range test for evaluating differences, essential prerequisites such as uniform variance and normal data spread were confirmed. Uniformity of variance was examined through Levene’s test, and normality was determined using the Shapiro–Wilk method. A *p*-value below 0.05 indicated statistical significance.

## 3 Results and analysis

### 3.1 Antagonistic effect of strain YL84 against FOV

**Figure 1:**
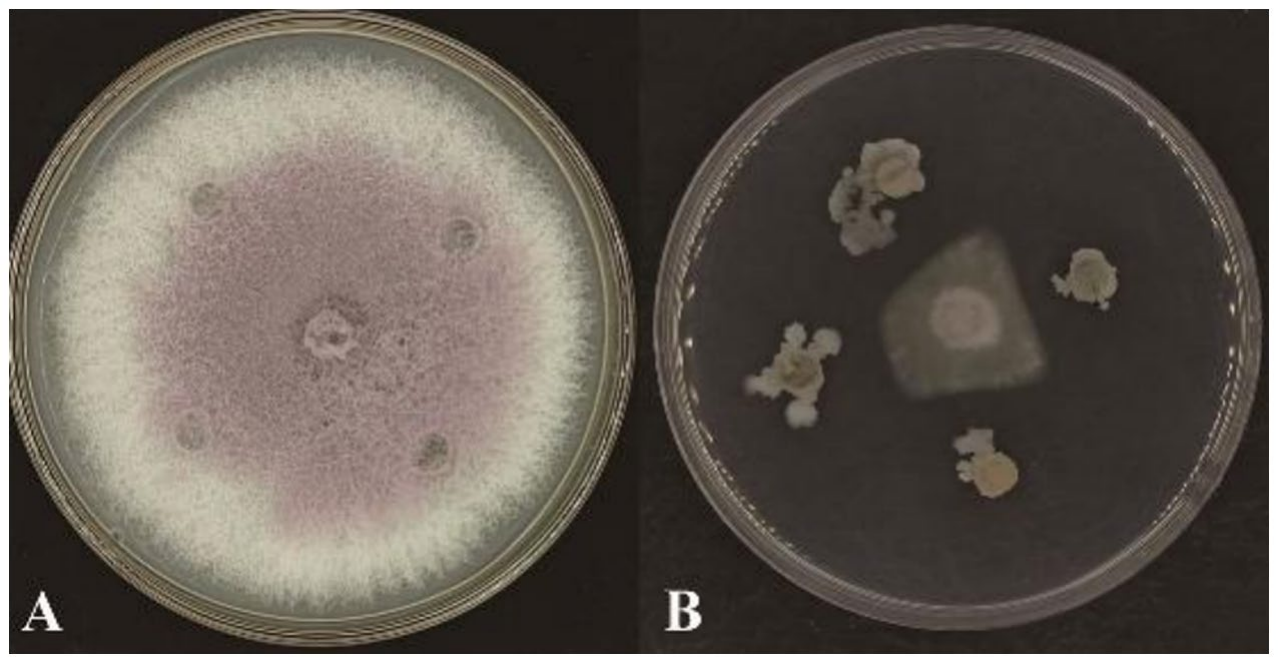
Antagonistic effect of strain YL84 against FOV. **(A)** Normal growth colony of FOV after 5 d; **(B)** Colony of FOV after 5 d of antagonism by YL84.

Plate confrontation assays demonstrated that strain YL84 exhibited strong antagonistic activity against FOV. After 5 d of incubation, the colony diameter of the pathogen in the control group reached 85 mm with dense mycelial growth (Figure 1A), whereas in the treatment group the average colony diameter was only 16.1 mm, with sparse mycelia (Figure 1B). The calculated inhibition rate of strain YL84 against FOV on plates reached 81.06%, confirming its remarkable antifungal efficacy.

### 3.2 Inhibitory effect of YL84 CFFF on conidial germination of FOV

**Figure 2:**
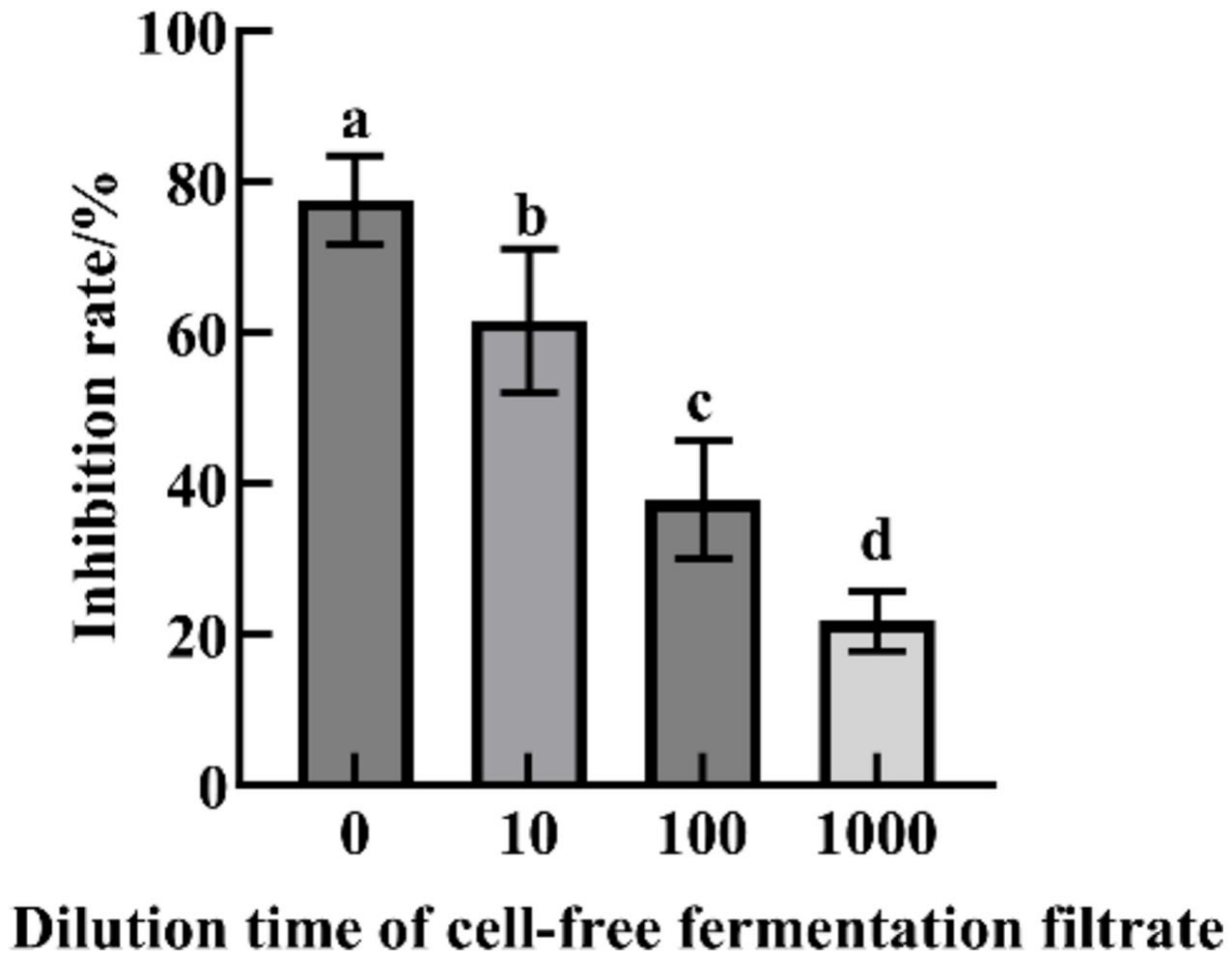
Inhibitory effect of cell-free fermentation filtrate on spore germination of FOV. Different lowercase letters indicate significant difference by Duncan’s new multiple range test (*p* < 0.05).

The CFFF of YL84 exhibited significant inhibitory activity against conidial germination of FOV in a concentration-dependent manner. The inhibitory effect gradually weakened with increasing dilution. The undiluted filtrate (dilution factor 0) showed the strongest effect, with an inhibition rate of 77.56%, whereas the 1,000-fold diluted filtrate had the lowest inhibition rate of 21.79% (Figure 2).

### 3.3 Inhibitory effect of YL84 CFFF on mycelial growth of FOV

**Figure 3:**
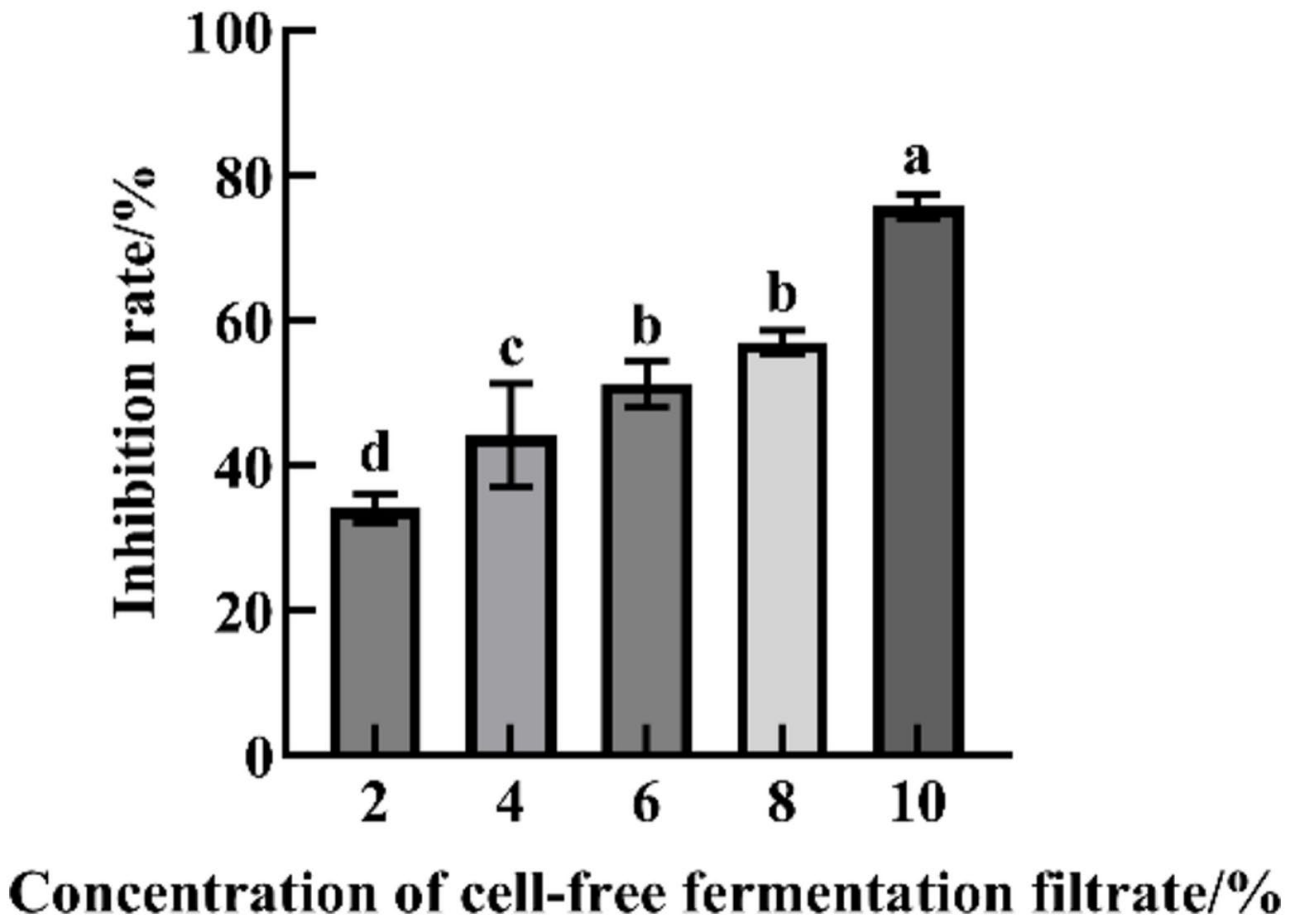
Inhibitory effect of cell-free fermentation filtrate on mycelial growth of FOV. Different lowercase letters indicate significant difference by Duncan’s new multiple range test (*p* < 0.05).

**Figure 4:**
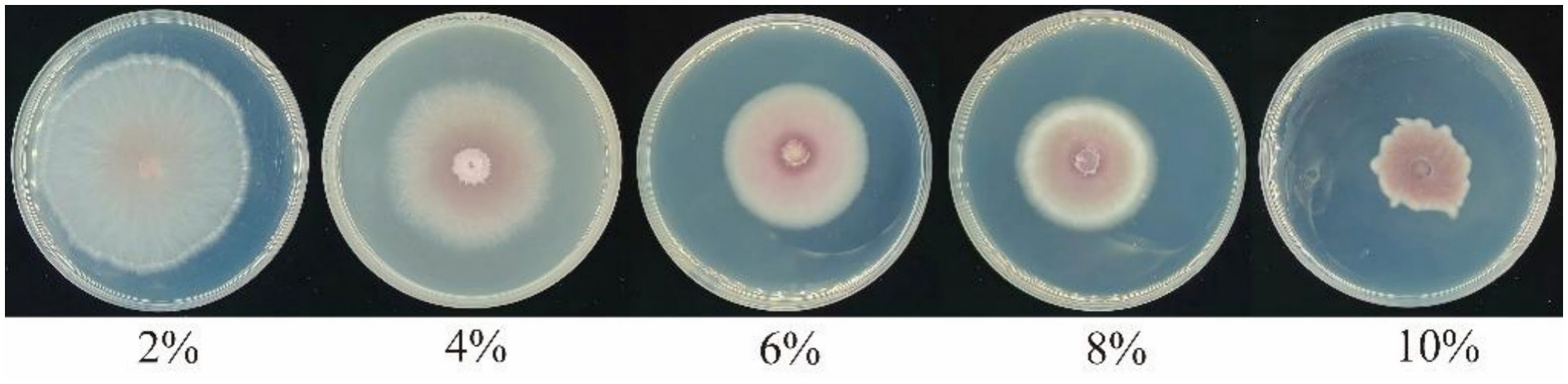
Effect of cell-free fermentation filtrate on mycelial growth of FOV.

The CFFF of strain YL84 effectively inhibited mycelial growth of FOV, and the inhibition rate was positively correlated with the volume fraction of the filtrate. Within the tested concentration range, the weakest inhibition occurred at 2% volume fraction (34.04%), while the strongest inhibition was observed at 10% volume fraction, with an inhibition rate of 75.68% (Figures 3, 4).

### 3.4 Effects of YL84 CFFF on nucleic acid leakage and malondialdehyde (MDA) content in FOV mycelia

**Figure 5:**
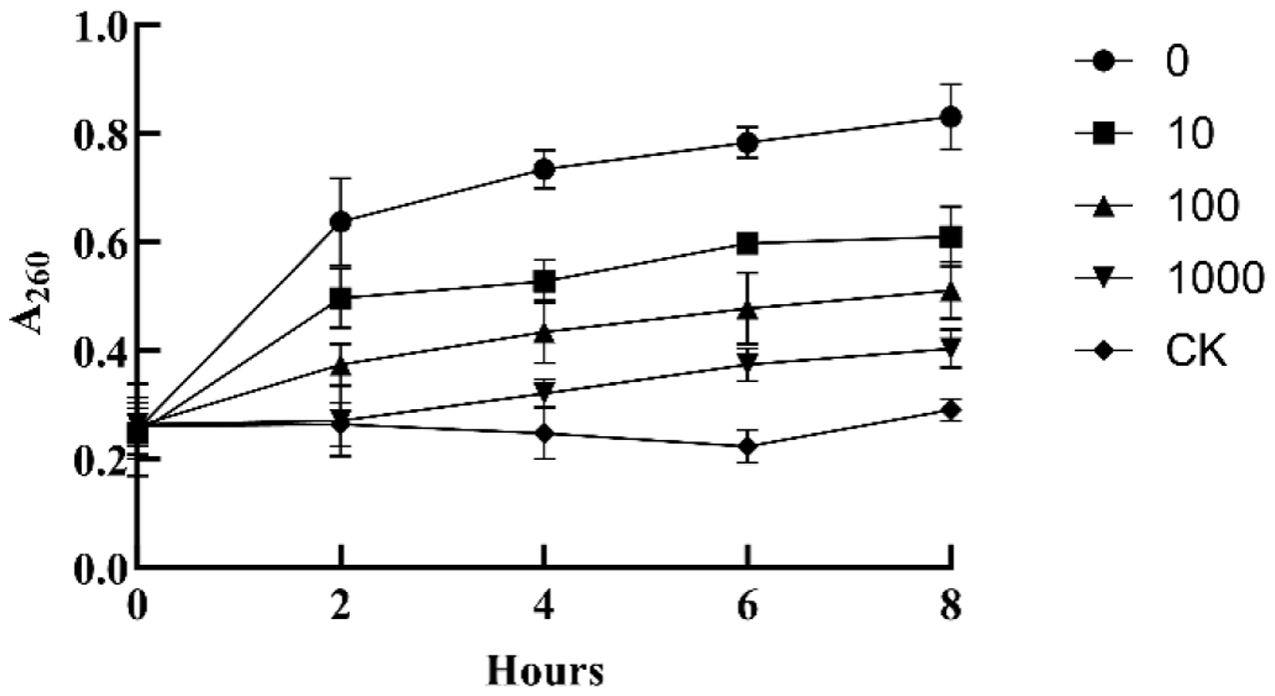
Effect of cell-free fermentation filtrate from strain YL84 on nucleic acid leakage from FOV mycelia.

**Figure 6:**
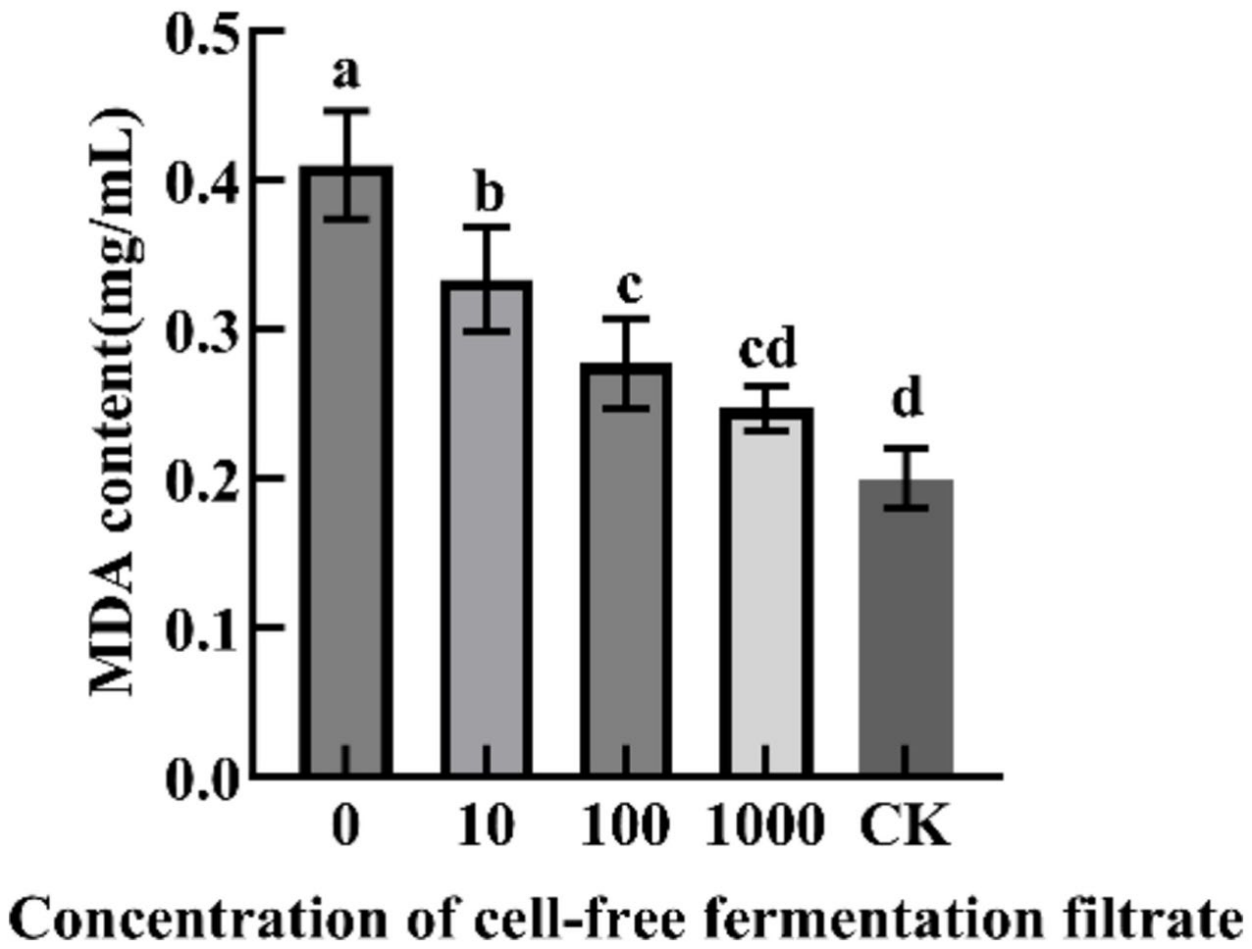
Effect of cell-free fermentation filtrate from strain YL84 on MDA content in FOV mycelia. Different lowercase letters indicate significant difference by Duncan’s new multiple range test (*p* < 0.05).

Treatment with CFFF significantly increased nucleic acid leakage from FOV mycelia. Absorbance at 260 nm (A260) peaked after 8 h of treatment, with the highest value in the undiluted group (0.83) and the lowest in the 1,000-fold diluted group (0.40) (Figure 5). After 24 h of treatment, MDA content in mycelia increased significantly, reaching 2.05 times that of the control in the undiluted group (Figure 6). These results indicate that YL84 CFFF disrupts the integrity of the pathogen’s cell membrane and induces oxidative damage.

### 3.5 Stability of the antifungal activity of YL84 CFFF

**Figure 7:**
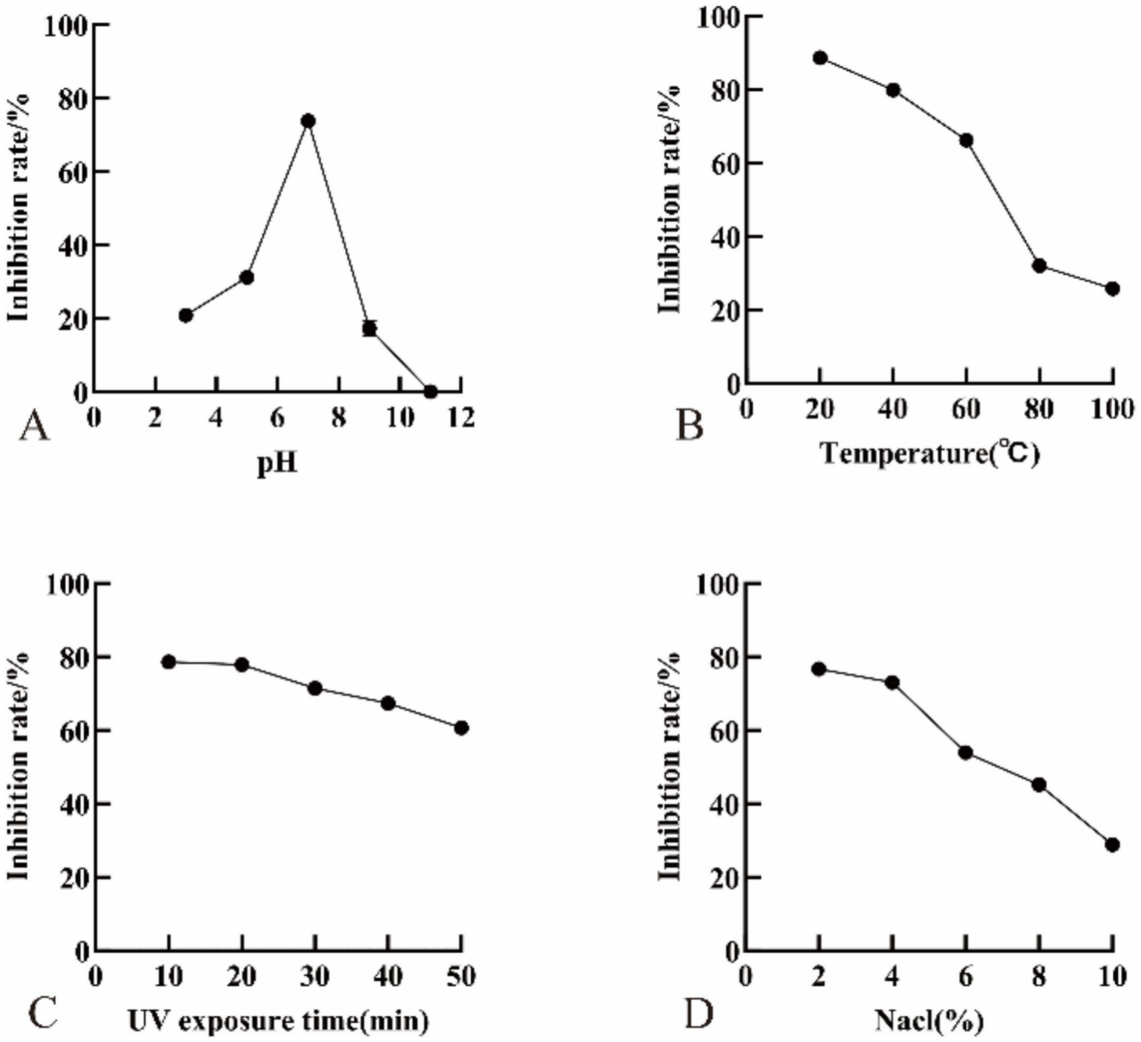
Antibacterial stability of the cell-free fermentation filtrate from strain YL84. **(A)** pH; **(B)** temperature; **(C)** UV exposure; **(D)** NaCl.

pH stability (Figure 7A): The CFFF exhibited the highest antifungal activity at pH 7.0 (73.53%). Under strong acidic conditions (pH 3.0), the inhibition rate dropped significantly to 20.58%, while at pH 11.0 the activity was completely lost, indicating certain tolerance to acid but sensitivity to strong alkali.

Temperature stability (Figure 7B): Antifungal activity decreased with increasing temperature, with optimal activity at 20 °C (88.47%). After treatment at 100 °C, 25.88% of the activity was retained, demonstrating good temperature stability.

UV stability (Figure 7C): UV irradiation for 10 min or 20 min had no significant effect on activity; even after 50 min, the inhibition rate remained at 59.65%, indicating good tolerance to UV radiation.

NaCl tolerance (Figure 7D): NaCl concentration significantly affected antifungal activity. The highest inhibition rate was observed at 2% NaCl (76.59%), and activity gradually decreased with increasing concentration, dropping to 28.82% at 10% NaCl, demonstrating moderate salt tolerance.

### 3.6 Control efficacy of YL84 CFFF against cotton *Fusarium* wilt in pot experiments

Pot experiments showed that YL84 CFFF exhibited good control efficacy against cotton *Fusarium* wilt (Table 2). Control efficacy was evaluated 21 d after root irrigation based on disease severity scoring and subsequent calculation of control efficacy. After 21 d of root irrigation, control efficacy decreased with increasing dilution, indicating a clear concentration-dependent response of the CFFF under greenhouse conditions. The undiluted filtrate achieved the highest efficacy of 69.21%, whereas the 1,000-fold diluted filtrate had the lowest efficacy of 24.62%.

**Table 2:** Control efficacy of strain YL84 cell-free fermentation filtrate against cotton *Fusarium* wilt in pot experiments.

Treatment	Disease index	Control efficiency/%
CK	86.67 ± 3.72 a	–
0	26.67 ± 2.53 e	69.21 ± 2.93 a
10	38.67 ± 3.43 d	55.38 ± 3.96 b
100	49.33 ± 2.83 c	43.08 ± 3.27 c
1,000	65.33 ± 2.33 b	24.62 ± 2.69 d

CK, inoculated control, plants inoculated with FOV but treated with sterile water (no CFFF); 0, plants treated with undiluted YL84 CFFF; 10, 100, and 1,000, plants treated with 10-fold, 100-fold, and 1,000-fold diluted YL84 CFFF, respectively. Different lowercase letters indicate significant difference by Duncan’s new multiple range test (*p* < 0.05).

### 3.7 Induction of resistance in cotton by YL84 CFFF

**Figure 8:**
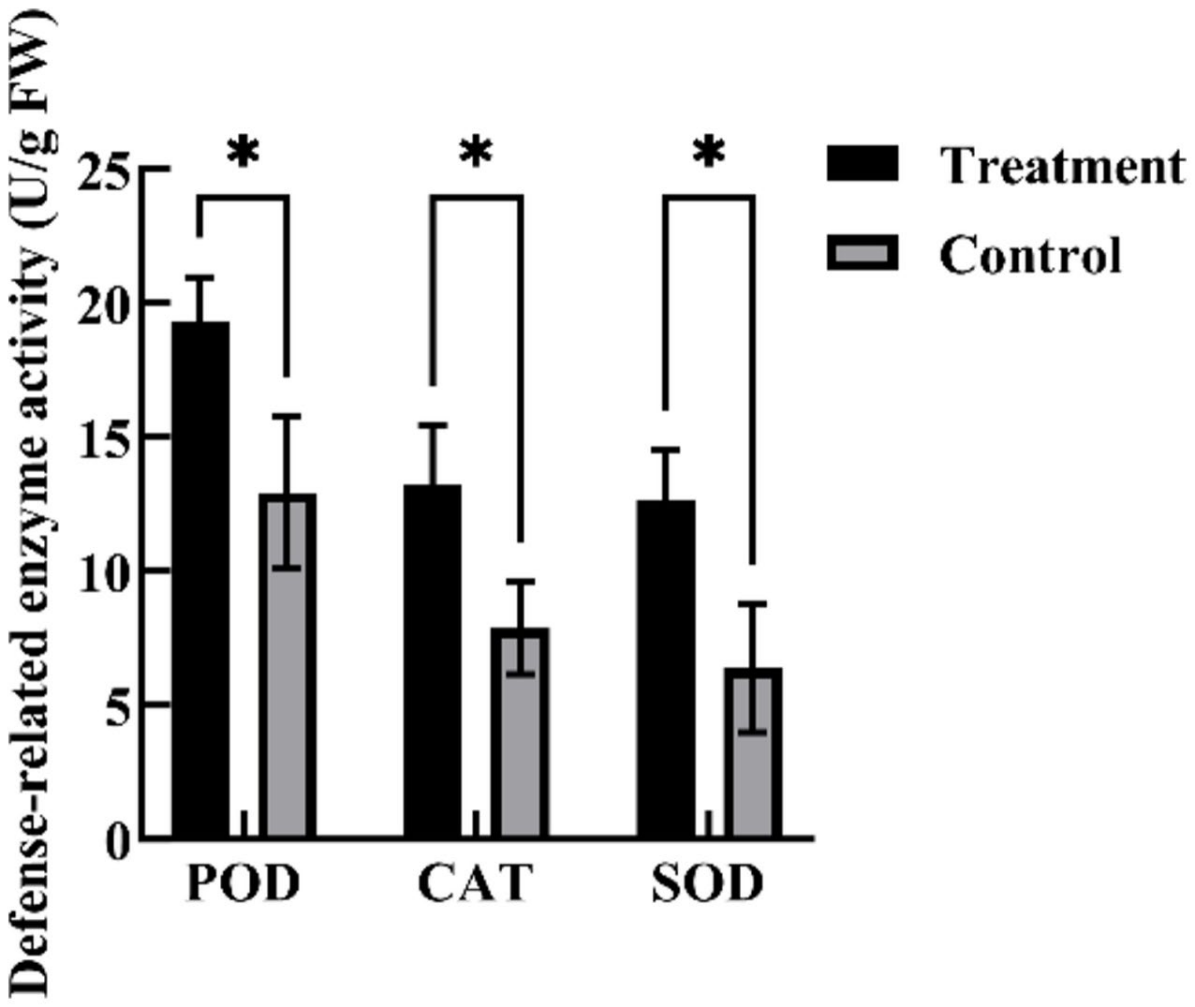
Effects of inoculation of antibacterial mixture culture on the defense reaction system of cotton. “*” indicates a statistically significant difference (*p* < 0.05).

Spraying YL84 CFFF significantly enhanced the activities of defense-related enzymes in cotton leaves (Figure 8). Compared with the control, POD, CAT, and especially SOD activities were significantly increased in treated plants, with SOD activity reaching 1.98 times that of the control. These results indicate that YL84 CFFF can induce systemic resistance in cotton plants by enhancing defense enzyme activities.

### 3.8 Component analysis of YL84 CFFF

**Figure 9:**
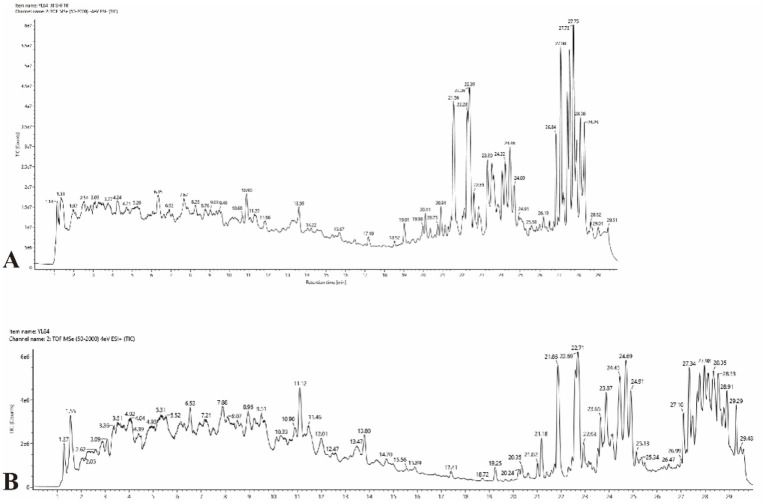
Total ion chromatograms (TIC) in positive and negative ion modes. **(A)** Negative; **(B)** positive.

Non-targeted metabolomics analysis using UPLC-IMS-Q-TOF-MS revealed a diverse metabolic profile in YL84 CFFF. Total ion chromatograms (TIC) in positive and negative ion modes (Figure 9) and identification results (Table 3) showed significant chemical diversity, including compounds putatively identified as diterpenoid glycosides (e.g., Pseudolaric acid-O-β-D-glucopyranoside), limonoids (e.g., Azedarachin C/E), diterpenoid lactone glycosides (e.g., Andrographolide 19β-glucoside), diterpenoid quinones (e.g., Tanshinone IIA/Hydroxytanshinone), benzophenanthridine alkaloids (e.g., Sanguinarine), lignans (e.g., Magnolol B), isoquinoline alkaloids (e.g., Epiberberine), phenolic acids (e.g., Gallic acid, Chlorogenic acid), and steroidal saponins (e.g., Polyphyllin IV). Compound annotation was based on high-confidence database matching of accurate mass, isotope distribution, retention time, and MS/MS fragmentation patterns (Supplementary material), without confirmation using authentic reference standards; therefore, all compound identities should be considered putative. This diverse chemical composition suggests that the antifungal activity may result from synergistic effects of multiple components rather than a single compound. In particular, membrane-active substances such as steroidal saponins (e.g., Polyphyllin IV) and certain diterpenoids are hypothesized to act as permeability enhancers, disrupting fungal cell membrane integrity and increasing permeability. This proposed mechanism provides a plausible explanation for the observed nucleic acid leakage and may facilitate transmembrane entry of other intracellular active substances (e.g., Sanguinarine and Tanshinone), thereby enhancing overall antifungal efficacy. However, further validation using purified compounds and standard-based confirmation is required to substantiate these mechanistic inferences.

**Table 3:** Identification of major potential antimicrobial components and their relative abundance in the cell-free fermentation filtrate of strain YL84 based on UPLC-IMS-Q-TOF-MS analysis.

No	Component	Adducts (+/−)	Response	Type	Neutral mass (Da)	Observed neutral mass (Da)
1	Pseudolaric acid-O-β-D-glucopyranoside	+	805,993	*Diterpenoid glycoside*	550.24141	550.2388
2	Azedarachin C/E	+	208,809/179,722	*Limonoid*	586.2778/738.36153	586.2754/738.3597
3	Andrographolide 19β-glucoside	+	184,049	*Diterpenoid lactone glycoside*	512.26215	512.2581
4	Tanshinone IIA/Hydroxytanshinone	−/+	14,776/8,528	*Diterpenoid quinone*	294.12559/310.12051	294.1219/310.1193
5	Sanguinarine	−	23,056	*Benzophenanthridine alkaloid*	331.08446	331.0840
6	Magnolol B	+	19,355	*Lignan*	280.10994	280.1062
7	Epiberberine	−	13,085	*Isoquinoline alkaloid*	335.11576	335.1114
8	Gallic acid	−	9,717	*Phenolic scid*	170.02152	170.0211
9	Polyphyllin IV	+	7,824	*Steroidal saponin*	854.46639	854.4644
10	Chlorogenic acid	−	15,556	*Phenolic Acid*	354.09508	354.0919

## 4 Discussion

The use of antagonistic microorganisms from natural environments for the control of plant diseases has become a research hotspot in the field of biological control (Pereg and McMillan, 2015; Rosenblueth and Martínez-Romero, 2006; Adhikari et al., 2021). Extensive studies, both domestically and internationally, have been conducted on the application of Bacillus spp. for the management of *Fusarium* wilt. For instance, Aslam et al. (2025) isolated 43 bacterial strains from the rhizosphere of diseased cotton plants, among which eight *Bacillus* strains exhibited inhibition rates against the pathogen ranging from 68.4% to 76.9%. Greenhouse experiments demonstrated that treatment with *B. subtilis* SC10 and SC11 significantly reduced the disease index by approximately 83%, with SC11 also showing vigorous plant growth-promoting potential, increasing shoot and root dry weight by 160% and 250%, respectively, while effectively enhancing antioxidant enzyme activities and alleviating oxidative damage. Yadav et al. (2021) screened 20 strains with broad-spectrum antagonistic activity against multiple pathogens from 118 rhizosphere isolates; strains EN21 and OR7 exhibited the highest *in vitro* inhibition rates (66.3%) against *Fusarium oxysporum* f. sp. *lactucae*. Greenhouse and field trials further confirmed that application of EN21 alone reduced disease severity by 44.91%–66.11%, while the combined application of EN21 and EN4 increased field control efficacy to 57.15%. Pengproh et al. (2023) isolated 11 *Bacillus* strains from soil, among which *Bacillus stercoris* B. PNR1 displayed broad-spectrum antifungal activity against *Fusarium oxysporum* f. sp. *lycopersici* and four other plant pathogenic fungi. Whole-genome sequencing revealed gene clusters associated with the synthesis of six classes of non-ribosomal peptides/polylactones, including bacilysin, surfactin, and bacillaene, which collectively mediate its biocontrol function. In the present study, *B. atrophaeus* YL84 was evaluated, and its CFFF demonstrated inhibition rates of 75.68% and 77.56% against mycelial growth and conidial germination of FOV, respectively, indicating considerable biocontrol potential. Furthermore, our findings suggest that YL84 CFFF may exert its biocontrol effects by disrupting fungal cell membrane integrity and inducing systemic resistance in cotton plants, as evidenced by increased nucleic acid leakage, elevated MDA levels, and enhanced antioxidant enzyme activities. However, it should be noted that these conclusions are based on indirect physiological indicators. The observed increase in membrane permeability could also result from non-specific stress responses or cell wall degradation, rather than direct membrane targeting. Similarly, while upregulated enzyme activities point toward induced resistance, confirming specific defense signaling pathways would require further molecular validation, such as gene expression profiling.

The stability of antifungal active substances is a critical factor determining their practical application potential in the field (Yan et al., 2024). Given the environmental characteristics of the central cotton-producing regions in Xinjiang—such as high temperatures, intense UV radiation, and soil salinization—antagonistic bacteria and their metabolites must exhibit certain tolerance to these extreme conditions. Barale et al. (2022) isolated *B. velezensis* SK from soil, and its fermentation products showed good thermal stability across a temperature range of 0–80 °C, maintaining high antibacterial activity against *Bacillus cereus* NCIM 2703 and *Staphylococcus aureus* NCIM 2654, particularly at 0, 20, and 37 °C; the products also remained stable within a pH range of 2–10. Similar conclusions were reported by Huang et al. (2023) and Ling et al. (2022). The results of this study indicate that the CFFF of *B. atrophaeus* YL84 retained substantial antifungal activity after exposure to high temperature, varying pH, UV irradiation, and different salt concentrations, confirming its tolerance to strong acid, high temperature, UV radiation, and salt stress.

UPLC-IMS-Q-TOF-MS technology integrates chromatographic separation, ion mobility spectrometry, and high-resolution mass spectrometry. This significantly reduces background interference from complex fermentation matrices, enabling accurate determination of known antagonistic compounds and effective identification of unknown active components (Costa et al., 2022; Tan et al., 2022). It has been widely applied in microbial metabolomics to explore biocontrol-related secondary metabolites and to generate hypotheses regarding potential modes of action. In the present study, non-targeted metabolomic analysis provided a comprehensive chemical profile of YL84 CFFF, in which multiple compounds were putatively identified based on high-confidence database matching, rather than confirmed by authentic standards. Based on this analysis, a range of putatively annotated metabolites belonging to different structural classes—including diterpenoids, alkaloids, phenolic acids, lignans, and steroidal saponins—were detected in YL84 CFFF. Although their identities remain putative, many of these compounds or their aglycones have been reported in previous studies to possess antifungal or antimicrobial activities. For example, the aglycone of Pseudolaric acid-O-β-D-glucopyranoside, pseudolaric acid, has been shown to exhibit strong antifungal activity (Zhang et al., 2017); Azedarachin C/E, a limonoid, is known for its inhibitory effects on pathogens (Da Silva et al., 2021); Andrographolide 19β-glucoside is related to andrographolide, which has well-documented antimicrobial and immunomodulatory properties (Kumar et al., 2020; Dafur et al., 2025). Similarly, Tanshinone IIA and Hydroxytanshinone have been reported to inhibit plant pathogenic fungi by disrupting cell membranes and inducing mitochondrial dysfunction (Ming et al., 2012; Zhang J. et al., 2018; Zhang P. et al., 2018), while Sanguinarine exhibits antifungal activity through membrane damage and reactive oxygen species accumulation (Huang et al., 2024). Phenolic acids such as gallic acid and chlorogenic acid, as well as steroidal saponins such as Polyphyllin IV, have also been reported to interfere with fungal growth through membrane- or oxidative stress–related mechanisms (Rhimi et al., 2020; Zhang et al., 2023). Taken together, these putatively identified metabolites may collectively contribute to the antifungal activity of YL84 CFFF, rather than the activity being driven by a single compound. In particular, membrane-active compounds such as steroidal saponins and certain diterpenoids are hypothesized to function as permeability enhancers, which may partially explain the observed nucleic acid leakage in FOV cells and potentially facilitate the intracellular entry of other bioactive components. Nevertheless, it should be emphasized that these interpretations are based on putative compound annotation and literature-supported functional inference; further validation using purified compounds and standard-based identification will be required to definitively establish their individual contributions and mechanisms of action.

## 5 Conclusion

In this study, *B. atrophaeus* YL84 was employed as the subject to evaluate the inhibitory effects of its CFFF against FOV systematically and to investigate its antifungal mechanisms preliminarily. The results demonstrated that the filtrate exhibited substantial inhibitory activity against mycelial growth and conidial germination of FOV. Following exposure to high temperatures, extreme pH values, UV radiation, and varying salt concentrations, the filtrate maintained high antifungal activity, highlighting its potential for application in the unique environmental conditions of cotton-producing regions in Xinjiang. Concurrently, this study confirmed the pot-based control efficacy of YL84 CFFF against cotton *Fusarium* wilt, as well as its role in inducing plant resistance. Furthermore, UPLC-IMS-Q-TOF-MS technology was utilized to conduct a comprehensive analysis of the antifungal active components in the filtrate. Future research will involve procuring corresponding standards for *in vitro* antifungal validation and conducting field trials further to elucidate the growth-promoting and control potential of strain YL84, thereby providing a robust theoretical foundation elucidated with specific candidate compounds and their stability profiles for the development of novel biological pesticides against cotton *Fusarium* wilt.

## Data Availability

The original contributions presented in this study are included in this article/Supplementary material, further inquiries can be directed to the corresponding authors.
